# Recent Advances in Mycotoxin Determination for Food Monitoring via Microchip

**DOI:** 10.3390/toxins9100324

**Published:** 2017-10-14

**Authors:** Yan Man, Gang Liang, An Li, Ligang Pan

**Affiliations:** 1 Beijing Research Center for Agricultural Standards and Testing, Beijing Academy of Agriculture and Forestry Sciences, Beijing 100097, China; manyan3669@163.com (Y.M.); liangg@brcast.org.cn (G.L.); lia@brcast.org.cn (A.L.); 2 Risk Assessment Lab for Agro-products, Ministry of Agriculture of the People's Republic of China, Beijing 100125, China; 3 Beijing Municipal Key Laboratory of Agriculture Environment Monitoring, Beijing 100097, China

**Keywords:** mycotoxin, microchip, microfluidic, microarray

## Abstract

Mycotoxins are one of the main factors impacting food safety. Mycotoxin contamination has threatened the health of humans and animals. Conventional methods for the detection of mycotoxins are gas chromatography (GC) or liquid chromatography (LC) coupled with mass spectrometry (MS), or enzyme-linked immunosorbent assay (ELISA). However, all these methods are time-consuming, require large-scale instruments and skilled technicians, and consume large amounts of hazardous regents and solvents. Interestingly, a microchip requires less sample consumption and short analysis time, and can realize the integration, miniaturization, and high-throughput detection of the samples. Hence, the application of a microchip for the detection of mycotoxins can make up for the deficiency of the conventional detection methods. This review focuses on the application of a microchip to detect mycotoxins in foods. The toxicities of mycotoxins and the materials of the microchip are firstly summarized in turn. Then the application of a microchip that integrates various kinds of detection methods (optical, electrochemical, photo-electrochemical, and label-free detection) to detect mycotoxins is reviewed in detail. Finally, challenges and future research directions in the development of a microchip to detect mycotoxins are previewed.

## 1. Introduction

Mycotoxins are a class of ubiquitious toxic compounds produced by the metabolism of certain fungi in food production [1,2]. Over 300 mycotoxins have been recognized and identified [3]. The most common mycotoxins are aflatoxins (AFs), alternaria toxins, ochratoxin A (OTA), deoxynivalenol (DON), zearalenone (ZEA), patulin (PTL), T-2 toxin (T-2), fumonisin B1 (FB1), and citrinin (CIT) [4]. The consumption of these mycotoxin-contaminated foods can cause severe toxic effects on human and animal health due to their mutagenicity, teratogenicity, carcinogenicity, nephrotoxicity, immunosuppression, and so on. They are more dangerous than food additives or pesticide residues [5]. Table 1 summarizes the representative mycotoxins and their toxicities. About 25% of food crops all over the world are significantly infected by mycotoxins according to the report of the Food and Agriculture Organization of the United Nations [6]. So far, there have been some statutory or guideline limits set for mycotoxins in food and feed by regulatory authorities worldwide; moreover, the regulated mycotoxins, commodities, and the maximum tolerable levels vary widely in different countries [7]. Hence, highly sensitive and reliable determination methods are requested in order to ensure food safety against mycotoxins.

Currently, numerous methods have been developed for the qualitative analysis of mycotoxins. Among these, liquid chromatography (LC) or gas chromatography (GC) coupled with an ultraviolet detector (UVD) [8,9], fluorescence detector (FLD) [10,11], or diode array detector (DAD) [12,13], or MS based on electrospray ionization (ESI) [14,15,16] or atmospheric pressure chemical ionization (APCI) [17,18,19] interfaces are the most common and preferred methods. Additionally, immunoassays are a powerful analytical technique that has been used extensively in the detection of mycotoxins [20,21,22]. Although these methods have high sensitivity and selectivity, they are laboratory-based, time-consuming, involve high costs, and often require large amounts of hazardous regents and solvents during the process of analysis.

Owing to its miniaturization, integration, automation, and high-throughput, an advanced microchip has attracted a large amount of interest as a rapid assay strategy in the detection of mycotoxins [23,24]. This work provides updated information on the determination of mycotoxins using microchips (both microfluidic chips and microarray chips). The materials used for the fabrication of microchip are briefly commented on. The emerging microchips based on optical detection, electrochemical detection, photo-electrochemical detection, as well as a label-free detection method are emphasized. Finally, the challenges and opportunities of using a microchip for the detection of mycotoxins in foods will be stated shortly.

## 2. Materials of the Microchip

Microchips, including microfluidic chips and microarray chips, are a powerful tool for realizing the quantitative analysis of a vast variety of objects with many advantages including high sensitivity, short analysis time, less sample and reagent consumption, high throughput, and low-cost analysis [58,59,60,61]. Microfluidic chips are the science and technology of systems that process or manipulate only 10^−9^ to 10^−18^ L of fluids, using channels with micro-sized dimensions [62,63,64]. Microarray chips are a two-dimensional arrangement of specific biological probes (DNA, polypeptides, proteins, cells, et al.) deposited in an accessible fashion on a glass slide or other substrate (polymer-coated glass, plastic, etc.) [65]. A vast variety of inorganic materials and polymers were used for the fabrication of microchips. Inorganic materials like silicon and glass are the first-generation materials used for the fabrication of microchips by photolithography and etching technology [62,66]. The chips based on silicon or glass substrates are widely used in capillary electrophoresis (CE) due to the stable electroosmotic mobility and high thermoconductivity. The major problem for silicon and glass chips is that the bonding is very difficult in the fabrication process due to the high temperature and super-clean lab environment that are usually required. In addition, the silicon is opaque. Hence, these limitations of the glass or silicon chips promote the development of polymer-based chips. The elastomeric polymer of polydimethylsiloxane (PDMS) has been the predominant polymer material for the preparation of microchips. Compared with glass and silicon chips, PDMS chips could be fabricated with the advantages of low cost, high elasticity, and easy and reversible bonding, and they could also be integrated with micropumps and microvalves. PDMS chips also have notable limitations, such as strong nonspecific adsorption and incompatibility with organic solvents. The other polymer chips were also accompanied by PDMS chips and have been identified as complementary to PDMS with the properties of rapid prototyping, higher rigidity, and better resistance to organic solvents [67]. For instance, rigid plastic polymers, such as poly(methyl methacrylate) (PMMA), polycarbonate (PC), polyimide (PI), polystyrene (PS), poly(vinyl chloride) (PVC), polyethylene terephthalate (PET), cyclic olefin copolymer (COC), teflon perfluoroalkoxy (PFA), and fluorinated ethylenepropylene (FEP) are the other types of materials for the fabrication of microchips. In addition, photosensitive polymers containing Norland optical adhesives (NOA81), SU-8 photoresist, and poly (ε-caprolactone (CL)-dl-lactide (LA)) (pCLLA) tetraacrylate, as well as hydrogel polymers, such as poly(ethylene glycol) (PEG), were also used for the fabrication of microchips. In recent years, paper substrates, which are widely available and inexpensive, have also been used for the fabrication of microchips. The advantages and disadvantages of the different microchip materials are summarized in Table 2.

## 3. Application of Microchips for the Determination of Mycotoxins in Foods

The detection methods integrated into microchips are mainly optical detection, electrochemical detection, photo-electrochemical detection, and a label-free detection method. Table 3 summarizes the representative microchips used to detect mycotoxins in food.

### 3.1. Optical Detection

#### 3.1.1. Fluorescence Detection

**Figure 1:**
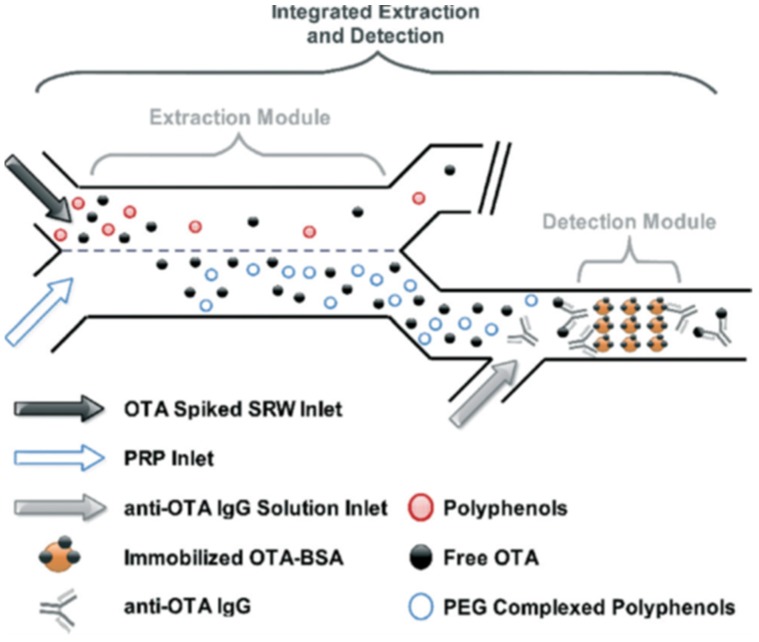
Conceptual schematic of the integrated microfluidic ATPE (aqueous two-phase extraction) strategy for matrix neutralization and OTA (ochratoxin A) concentration in red wine samples [22]. (Reprinted from reference [22], Copyright (2014), with permission from Royal Society of Chemistry).

Fluorescence detection has been widely used in the majority of work on microchips due to its excellent sensitivity [85]. In the detection of mycotoxins, Soares et al. [22] developed an integrated PDMS microchip device consisting of two modules in series, an aqueous two-phase extraction (ATPE) module for the simultaneous matrix clean-up, concentration of OTA in red wine and an indirect competitive fluorescence-linked immunosorbent assay module for OTA quantitative detection (Figure 1). The limit of detection (LOD) of OTA spiked in raw red wine was 0.26 μg/L. The novel microfluidic chip based on ATPE can deal with complex sample matrices without extra sample pretreatment processes and instruments. The microfluidic chip-based microarray analytical method by fluorescence detection also used for the detection of mycotoxins. Hu et al. [86] prepared a PDMS-glass microfluidic chip and formed a smectite–polyacrylamide (PAM) nanocomposite strips microarray on the glass slide by the layer-by-layer assembly method in the PDMS channel, for the rapid fluorometric quantitative detection of AFB1 in corn with an LOD of 6.09 μg/kg. Compared with the analysis of microarray chip, this method reduced the detection time, increased detective sensitivity, and enhanced analytical performance due to the parallel multi-channel.

**Figure 2:**
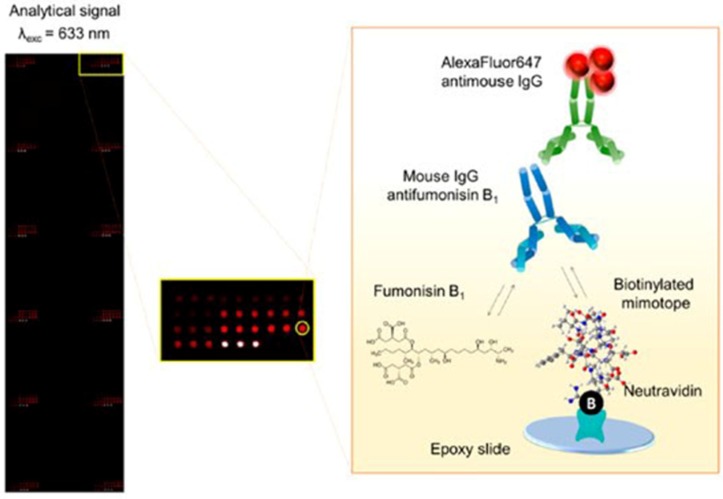
Schematic presentation of the microarray-based immunoassay for fumonisin detection with biotinylated mimotopes [89]. (Reprinted from reference [89], Copyright (2017), with permission from American Chemical Society).

In general terms, the synthesis of a mycotoxin and carrier protein conjugate is time-consuming, consumes large amounts of expensive organic reagents, and forms randomly crosslinked and unstable molecules, so that can be considered as one major drawback of the competitive immunoassay [87,88]. Nevertheless, a mimotope, as an alternative to a mycotoxin-protein conjugate, can be applied to competitive immunoassay. Peltomaa et al. [89] selected a novel mimotope from a commercial peptide library by phage display technology and, furthermore, developed a microarray immunochip using the synthetic derivative of this mimotope for the detection of FB1 in spiked maize and wheat with the LOD of 11.1 μg/L (Figure 2). The mimotope-based immunoassay showed similar or superior sensitivity and reproducibility, reduced analysis time and consumption of toxic reagents, and avoided the chemical synthesis of hapten and a hapten carrier complex.

Additionally, considering that a wide variety of mycotoxins have been found in foods, the detection of multiple toxic mycotoxins is of importance in food quality control, so it is urgent to develop multiplexed, high-throughput methods for mycotoxins detection. For instance, Miriam et al. [90,91] developed a competitive microarray immunochip for the simultaneous detection of OTA and DON. Biotinylated OTA and DON were immobilized in arrays onto the NeutrAvidin-functionalized waveguide, respectively. The LODs of OTA by fluorescence detection were 3.8–100 μg/kg in several cereals, 7 and 38 μg/kg in coffee and wine, respectively, and the LODs of DON were 0.2–50 μg/kg in oats and 4 μg/L in air effluents, respectively. Wang et al. [92] established a protein microarray immunochip for simultaneous and rapid detection of AFB1, AFM1, DON, OTA, T-2, and ZEA mycotoxins in drinking water. Six complete antigens were contact printed and immobilized onto agarose-modified glass chip, and a fluorescence indirect competitive immunoassay was used for the detection. The LODs of AFB1, AFM1, DON, OTA, T-2, and ZEA in drinking water were 0.01, 0.24, 15.45, 15.39, 0.05, and 0.01 μg/L, respectively.

Furthermore, an aptamer can be used as an alternative to an antibody in the development of biosensors and other analytical methods due to its unique features such as good stability, easy modification, and low-cost production. Yang et al. [93] designed a new high-throughput biochip based on photonic crystal microsphere (PHCM) suspension array for the detection of AFB1, OTA, and FB1 mycotoxins in cereal samples. The hybridization duplex strands of toxin aptamers and their partial complementary strands were immobilized on the surfaces of silica PHCMs. When toxins are present in the sample, the aptamers will specifically bind to their target mycotoxins, the distance between fluorescent dye and quencher increases, and the fluorescence signal recovers. The LODs were 15.96 × 10^−6^ μg/L for AFB1, 3.96 × 10^−6^ μg/L for OTA and 0.011 μg/L for FB1.

To conclude, although the abovementioned fluorescence detection methods show good sensitivity and reproducibility, and the labeled molecules are small in size, they require external light sources or the excitation of fluorescent labels, so the integrated optical components are complicated, expensive, large, and bulky. Moreover, the detection signal is also susceptible to interference from scattered light, background fluorescence, and turbidity of samples.

#### 3.1.2. Chemiluminescence Detection

Chemiluminescent detection has been used in the analysis of microchips due to the advantages of low detection limits and wide dynamic ranges, and, compared with fluorescence detection, chemiluminescent detection only requires simple instrument configuration as a result of no external light sources or spectroscopes [94,95]. Sauceda-Friebe [96] developed an indirect competitive immunochip and prepared a fully automated flow through the device with a chemiluminescence readout system for the detection of OTA in green coffee extract. The peptide-linked OTAs were synthesized and covalent immobilized to the epoxy derivatized glass chip surface. The peptide linker provides free amino groups available for the covalent coupling of compounds and supplies a biocompatible space for the target analyte. The chemiluminescence signals were collected with only a CCD camera, and the limit of quantitation (LOQ) of OTA in green coffee extract was 0.3 μg/L. Moreover, this method has the potential for on-site detection due to all the steps of experiment being automated. Furthermore, Oswald et al. [97] developed a regenerable, reusable glass microarray chip for the parallel detection of AFB1, AFB2, OTA, DON, and FB1 multiple mycotoxins in oat extracts by a fully automated chemiluminescence microarray readout device. However, the emission intensity of chemiluminescent detection depends on a variety of environmental factors such as temperature, pH, ionic strength, and so on [95]. Hence, the environmental factors should be strictly controlled in the detection process.

#### 3.1.3. Colorimetric Detection

**Figure 3:**
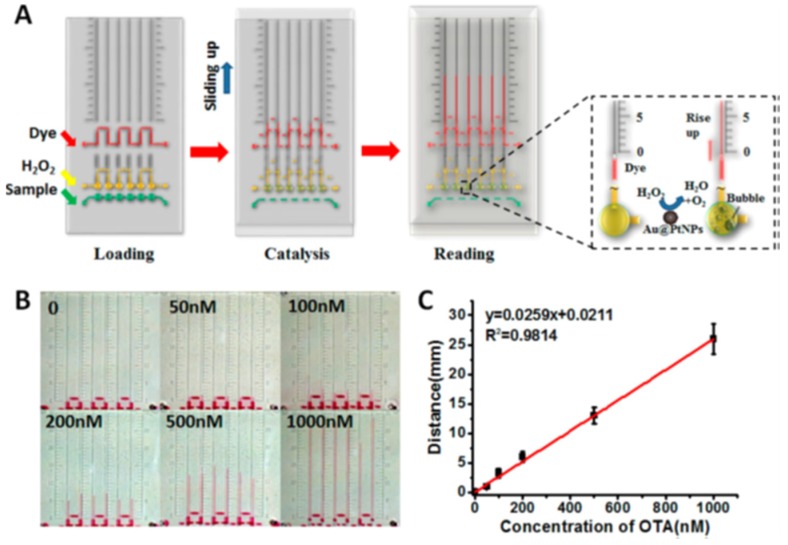
Performance of the HV-Chip for the detection of OTA [98]. (**A**) Working principle of the target-responsive hydrogel combined with the volumetric bar-chart chip readout for visual quantitative detection. (**B**) Images showing ink advancement for the detection of OTA in the range of 0 to 1000 nM in 30 min. (**C**) Linear standard curve was obtained from 0 to 1000 nM OTA. (Reprinted from reference [98], Copyright (2015), with permission from American Chemical Society).

Recently, a rapid, portable, and inexpensive detection method without the need for sophisticated instrumentation was developed for mycotoxin detection by visual colorimetric analysis. Liu et al. [98] designed and synthesized an OTA-responsive aptamer-cross-linked hydrogel and used it to fabricate a volumetric bar chart chip (V-chip) for portable and visual quantitative detection of OTA in beer (Figure 3). The OTA responsive hydrogel was prepared by the mixture of OTA aptamer, polymer strands A (P-SA), polymer strands B (P-SB), and platinum nanoparticles (PtNPs). Here, P-SA and P-SB were obtained by grafting short DNA strands A and B onto polyacrylamide polymers. Moreover, the OTA aptamer is complementary to short strands A and B. The OTA in beer was detected by the prepared V-chip, and the LOD was 0.51 μg/kg. Ma et al. [99] also applied the same approach for the detection of AFB1 in beer with the LOD of 0.55 μg/kg. Thus, the portable, visual, and quantitative detection of a target in real samples can be achieved by a distance-based readout V-chip combined with a target-responsive hydrogel. Additionally, Li et al. [100] developed an integrated smartphone-app-chip (SPAC) system for the quantitation of AFB1 in corn samples with LOD of 3 μg/kg. The detection was realized on a transparent plastic chip plate by an indirect competitive immunoassay. A cost-effective 3D-printed optical accessory attached to a smartphone was used to provide uniform illumination for imaging the chip, then the chip images were captured by the smartphone camera and directly processed by an Android app. The SPAC system was suitable for real-time, on-site detecting of AFB1 in foods in China and North America. Colorimetric detection provides high sensitivity, selectivity, low cost, and portability without any large-scale instrument, so it is suitable for rapid, real-time, on-site quantitative detection of mycotoxins to ensure food safety.

### 3.2. Electrochemical Detection

The properties of high sensitivity and selectivity, the response not being limited by optical path length and sample turbidity, high compatibility, miniaturization, and integration have enabled electrochemical detection method to become an excellent technique to incorporate into microchip devices [101,102]. Piermarini et al. [103] prepared 96-well screen-printed carbon electrodes, and incorporated them in the electrochemical plate. Then the 96-well screen-printed microarray chip was used for an indirect competitive immunoassay by electrochemical detection for the detection of AFB1 in corn. An AFB1–bovine serum albumin (BSA) conjugate was immobilized on the carbon electrodes by physical absorption. The LOD of AFB1 was 0.03 μg/L; the working range was 0.05–2 μg/L. For the carbon electrodes, Parker et al. [104] fabricated a gold electrode microarray immunochip by deposition, etching, and lithographic techniques for the detection of AFM1 in milk. AFM1 antibodies were immobilized on the gold microelectrodes by chemical cross-linking with 1,4-phenylene diisothiocyanate. A competitive electrochemical immunoassay was developed on the prepared gold microelectrode surface. The LOD of AFM1 in milk was 0.008 μg/L. Compared with abovementioned screen-printed carbon electrodes, the gold microelectrodes can offer a steady-state cyclic voltammogram and a higher sensitivity.

**Figure 4:**
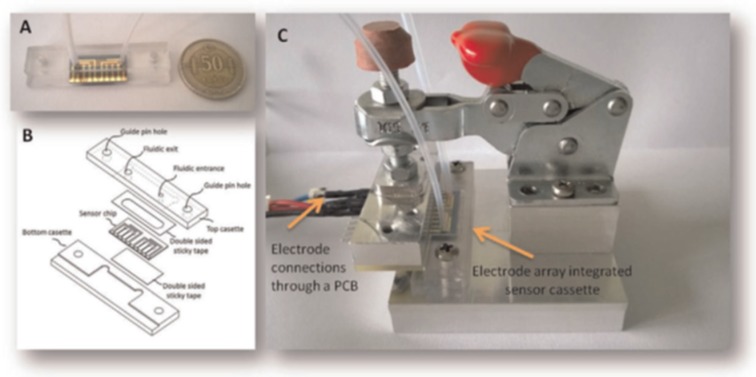
(**A**) Electrode array integrated sensor cassette. (**B**) A flow cell is formed and the electrode array is fixed to the sensor cassette by means of a double sided sticky tape. (**C**) For the laboratory prototype, a printed circuit board (PCB) attached clamp is used to establish electronic connections between the electrode arrays and the potentiostat device [106]. (Reprinted from reference [106], Copyright (2014), with permission from Elsevier).

Additionally, a microfluidic immunochip was developed by Arévalo et al. [105] for the detection of CIT in rice by electrochemical detection; CIT-ovalbumin (OVA) conjugates were immobilized on gold disk modified with cysteamine, the LOD of 0.1 μg/L and LOQ of 0.5 μg/L for CIT. Furthermore, the microfluidic chip coupled with gold microelectrode arrays was developed by Olcer [106] and Uludag [107] for real-time electrochemical detection of DON in wheat with a LOD of 6.25 μg/L and AFB1 in foods with a LOD of 0.08–0.65 μg/kg (Figure 4). The novel biochips consisted of six working electrodes with shared references and counter electrodes, and the microfluidic channel was created on the electrode arrays by double-sided sticky tape. The real-time electrochemical microfluidic chip would provide an on-site, rapid, and cost-effective solution for the detection of mycotoxins. 

Electrochemical microfluidic chips coupled with magnetic bead-based immunoassay were also used to determine mycotoxins. Hervás [108,109] and Panini [110] integrated magnetic bead-based electrochemical enzyme-linked immunosorbent assay (ELISA) on microfluidic chips for the determination of ZEA. The ZEA in real samples and HRP–ZEA conjugates compete for the binding sites of anti-ZEA monoclonal antibodies, which were immobilized on protein G modified magnetic beads. The LODs of ZEA in baby foods and feed samples were 0.4–1 μg/L. Moreover, this method was also used by Fernández-Baldo for the detection of OTA in apples with a LOD of 50 μg/kg [111]. Here, magnetic beads acted as antibody immobilization carriers and the mobile substrates in microfluidic chips can be easily handled and manipulated by an external magnet. Moreover, due to the use of magnetic beads, the performance of the immunoassay was improved as a result of the increased surface-to-volume ratio.

### 3.3. Photo-Electrochemical Detection

Photosensors, especially those based on hydrogenated amorphous silicon (a-Si:H), have been widely integrated into microchips for low-cost, real-time, highly sensitive, miniaturized analysis due to the advantages of optimal optical coupling, good signal-to-noise ratio, and low deposition temperature (below 250 °C), which allows for the use of glass, plastic, and polymer substrates [112,113,114]. Novo et al. [115] developed an indirect competitive ELISA in a PDMS microfluidic chip with integrated a-Si:H photodiode arrays for the chemiluminescence detection of OTA in red wine and beer. OTA–BSA was immobilized into the microchannel inner surface by physical absorption. Compared with a straight-channel configuration, the PDMS microfluidic chip with a two-channel U-shaped configuration can significantly reduce measurement errors, and the LODs of 0.1 and 2 μg/L for beer and red wine, respectively. In addition, Soares [116] developed a regenerable microfluidic immunochip with integrated photosensors for the detection of OTA in red wine. The regeneration protocol was achieved using a glycine solution (pH = 2) to selectively disrupt the interaction between antigen and antibody, and the PDMS chip could be effectively used at least eight times.

Simultaneous detection of multiple mycotoxins by a microfluidic a-Si:H photosensors was also realized. Soares et al. [117] developed a microfluidic multiplexed immunochip utilizing a permanent magnet valve and a single negative pressure source for the simultaneous detection of OTA, AFB1, and DON (three mycotoxins) by the integrated a-Si:H photodetector. Three mycotoxins of 100 μg/L for OTA and DON, and 3 μg/L for AFB1 were detected in less than 20 min. Apart from the abovementioned immunosensors, an aptasensor has also been developed in a microfluidic chip with integrated a-Si:H photodiode arrays for mycotoxin detection. Costantini et al. [118] developed an aptamer-based sandwich assay (ALISA) in a multichannel microfluidic chip with an array of a-Si:H photosensors for OTA detection. Aptamer-linked amino was immobilized on a glass substrate modified with succinic anhydride by a condensation reaction. When OTA present in the sample, an aptamer sandwich-like structure will be formed and the generated chemiluminescent signal will be detected by a-Si:H photosensors. The LOD and LOQ of OTA in beer were 0.82 and 2.5 μg/L, respectively.

### 3.4. Label-Free Detection

#### 3.4.1. MS Detection

The microfluidic chips integrated with LC-MS can reduce matrix interference, sample consumption, and dead volume, improve detection sensitivity, and realize online sample pre-concentration. Jiang et al. [119] fabricated a plastic microfluidic chip and coupled it with ESI-MS for AFB1 detection. The copolyester plastic chip was constructed by a silicon template imprinting technique. The detection sensitivity was increased by 1-2 orders of magnitude compared to the previously reported LC-MS method [120]. Recently, Liu et al. [121] employed a microfluidic chip-based nano LC coupled with a triple quadrupole mass spectrometer (QqQ-MS) system for the quantitative detection of AFs (AFB1, AFB2, AFG1, AFG2, and AFM1) in peanut products. The samples were treated successively by solvent extraction and immunoaffinity SPE for the purification and concentration of target molecules. The LODs were 0.004–0.008 μg/kg and the linear range was 0.048–16 μg/kg. The sensitivities of chip-nanoLC-tandem MS were improved about 10 times compared to the conventional HPLC-MS/MS method.

#### 3.4.2. Surface Plasmon Resonance (SPR) Detection

SPR has already attracted extensive attention as a label-free method for the detection of mycotoxins due to the short analysis time, simple and rapid cleanup procedures, and reusability [122,123]. SPR is used to monitor the intermolecular interactions on a biosensor chip’s surface, such as the interactions of antibodies, aptamers, or molecularly imprinted polymers (MIPs) with the target molecules.

##### Microchip SPR Sensors Based on Antibodies

In order to realize the detection of mycotoxins by microchip SPR immunosensors, highly specific single-chain variable antibody fragments (scFvs) of AFB2 were firstly prepared by Edupuganti et al. [124]. Then the microchip SPR sensor based on competitive inhibition immunoassay was developed using scFv-E9 for the detection of AFB2 in a spiked almond sample with an LOD of 0.9 μg/L. Then they also generated an anti-ZEA scFv antibody, and used the same approach for the detection of ZEA in sorghum [125]. In addition, Kadota et al. [126] developed a microchip SPR immunosensor using a monoclonal antibody for rapidly measuring the sum of DON and NIV in wheat. The monoclonal antibody cross-reacts with DON and NIV. DON and NIV were purified using an immunoaffinity column, and the recoveries were 91.5%–107%. The LODs were 50 μg/kg and 100 μg/kg for DON and NIV, respectively. The results of microchip SPR sensor were correlated with those obtained using LC/MS-MS. T-2 and HT-2 toxins in cereals were also detected using the same method by Meneely et al. [127]. The LODs were determined as 25 μg/kg for baby food and breakfast cereal, and 26 μg/kg for wheat. Then they also used the SPR sensor for the simultaneous determination of the sum of T-2, HT-2, and DON in cereals [128]. LODs of 12, 1, and 29 μg/kg for DON and 31, 47, and 36 μg/kg for HT-2 were found in wheat, breakfast cereal, and maize-based baby food, respectively. Moreover, PTL toxin was also detected by the microchip SPR sensor using the produced polyclonal mono-specific antibodies, which were coated on the sensing interface, and the LOD was 15.41 μg/L [129].

**Figure 5:**
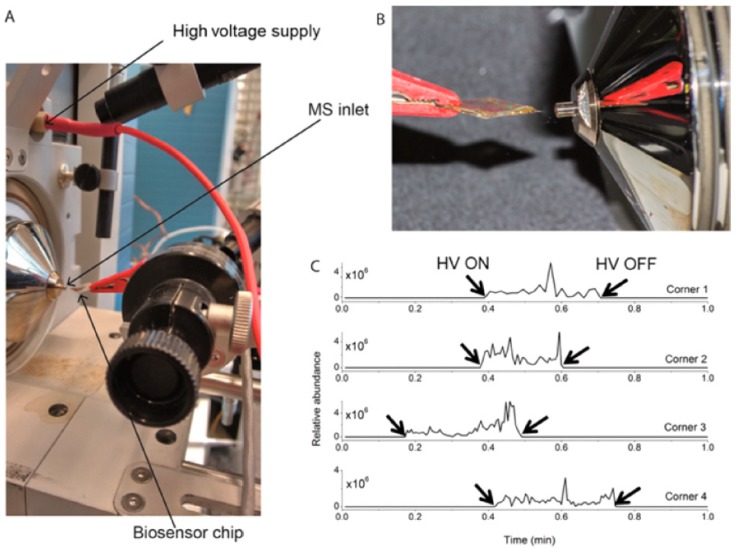
(**A**) Setup used for Biochip Spray MS using a gold biosensor chip held in front of the MS inlet using an alligator clip and (**B**) spray obtained after adding 10 μL of methanol and applying a voltage of 5 kV. (**C**) Extracted ion chronogram for *m/z* 297.1333 ([DON + H]^+^) recorded in positive ion mode, as obtained from four different corners of a single 1 cm^2^ square carboxymethylated dextran (CMD) modified gold chip [133]. (Reprinted from reference [133], Copyright (2017), with permission from American Chemical Society).

Performing SPR in an imaging format (iSPR) allows for high-throughput detection of mycotoxins. For example, Dorokhin et al. [130] applied a multiplex microassay iSPR immunochip for rapid screening of DON and ZEA. DON–OVA and ZEA–OVA conjugates were immobilized on the carboxylated chip surface. The LODs were 84 and 68 μg/kg for DON and 64 and 40 μg/kg for ZEA in maize and wheat samples, respectively. Joshi et al. [131] prepared a portable nanostructured iSPR immunochip for the analysis of DON and OTA in beer with LODs of 17 μg/L for DON and 7 μg/L for OTA. The authors also developed a benchtop SPR (Biacore) with two separate nanostructured iSPR immunochip for the portable and multiplex detection of DON, ZEA, T-2, OTA, FB1, and AFB1 in barley [132]. The LODs were 26 μg/kg for DON, 6 μg/kg for ZEA, 0.6 μg/kg for T-2, 3 μg/kg for OTA, 2 μg/kg for FB1, and 0.6 μg/kg for AFB1. Additionally, they coupled SPR immunochip and ambient ionization MS for the detection of DON in beer (Figure 5) [133]. The coupling of SPR with MS could not only identify the SPR-detected target analytes, but also could distinguish the cross-reacting analytes.

In order to improve the detection sensitivity of iSPR immunochip, Hu et al. [134] designed a gold-nanoparticle-enhanced iSPR chip for simultaneous detection of AFB1, OTA, and ZEA mycotoxins in spiked peanut samples. The sensing interface was constructed by attaching the antigens of mycotoxins on poly[oligo(ethylene glycol)methacrylate-*co*-glycidyl methacrylate] (POEGMA-*co* GMA) brush modified iSPR gold chip. AuNPs–secondary antibody conjugates were combined with the captured monoclonal antibody for further amplification of the iSPR signal. The iSPR chip shows high sensitivity and specificity for AFB1, OTA, and ZEA, with LODs of 0.008, 0.03, and 0.015 μg/L, respectively. Moreover, Karczmarczyk et al. [135,136] also utilized the gold-nanoparticle-enhanced SPR immunochip for the detection of OTA in red wine and AFM1 in milk; the LODs were 0.068 μg/L for OTA and 0.018 μg/L for AFM1. 

However, the immobilization of antibodies on the sensing interface by physical adsorption or covalent binding can lead to instability and loss of binding capacity in antibodies. An effective method was constructed by Park et al. [137] for immobilizing antibodies on the sensing surface. They firstly produced a gold binding protein (GBP)–protein G (ProG) bifunctional crosslinker by genetic engineering, and then used the GBP–ProG crosslinker for the immobilization of antibodies on chip gold substrates without any chemical treatment. The fabricated SPR chips based on the GBP–ProG crosslinker were used for the detection of AFB1 in both buffer and corn extracts with the LOD of 1000 μg/L.

##### Microchip SPR Biosensors Based on Aptamers

In recent years, microchip SPR biosensors based on aptamers also have been applied to the detection of mycotoxins. Zhu et al. [138] established a SPR biosensor chip using anti-OTA aptamers for quantitative detection of OTA in wine and peanut oil. The streptavidin was immobilized onto the chip surface with dextran matrix using an amine coupling method; the biotin–aptamer was captured by the interaction of streptavidin and biotin. The SPR biosensor gives high sensitivity with an LOD of 0.005 μg/L and a linear range of 0.094–10 μg/L. AFB1 in red wine and beer was also detected using the same approach, with an LOD of 124.91 μg/L, by Sun et al. [139].

A localized SPR (LSPR) biosensor system formed by the immobilization of colloidal metal nanoparticles on surfaces has several advantages, such as device miniaturization, regeneration and reuse, and multiplex detection. Park et al. [140] prepared a LSPR aptasensor chip for the detection of OTA in ground corn samples. OTA aptamer immobilized on the surfaces of gold nanorods (GNRs) will self-assemble into a G-quadruplex structure when it interacts with OTA, and the LOD was below 403 μg/L. This LSPR biosensor chip was first developed for the detection of small molecules. Additionally, Bianco et al. [141] developed an aptamer-based SPR-polarization biosensor chip for the detection of OTA, with an LOD of 0.005 μg/L. The phase-interrogation SPR biosensor chip was found to be a highly sensitive detection technique with potential use as portable device.

##### Microchip SPR Biosensors Based on MIP

Compared to the antibody and aptamer, MIP is a synthetic recognition element that can remain stable in harsh conditions, such as high temperature and pressure, extreme pH, and even organic solvents. MIP technique is an effective method in terms of specific recognition. Hence, microchip SPR biosensors based on MIP have many advantages including high specificity, sensitivity, and selectivity. Choi et al. [142] fabricated a SPR biosensor chip based on MIP for the detection of DON. An MIP film of 5 nm thickness was prepared on a bare Au chip using pyrrole by electropolymerization when DON was a template. The SPR biosensor chip exhibited a linear response in the range of 0.1–100 μg/L for a DON standard solution, and the LOD was about at >1 μg/L. Moreover, the authors also detected ZEA in corn using this method, with an LOD of 0.3 μg/kg [143]. Moreover, Atar et al. [144] prepared CIT-imprinted poly(2-hydroxyethyl methacrylate–methacryloylamidoglutamic acid) (p(HEMA–MAGA)) film on the gold surface of SPR chip for the detection of CIT in red yeast rice. The linearity range was 0.005–1.0 μg/L and the LOD was 0.0017 μg/L. In addition, Gupta et al. [145] prepared nanopatterned π-conjugated MIP on a SPR chip for supersensitive detection of T-2 by in situ electropolymerization of T-2 with 3-aminophenylboronicacid (3-APBA). The developed method showed a linear response from 0.98 × 10^−3^ to 0.016 μg/L for T-2, and the LOD was 0.47 × 10^−4^ μg/L. In conclusion, the microchip SPR biosensor chip based on MIP is a sensitive, rapid, cheap, robust, and easy method for the detection of mycotoxins.

#### 3.4.3. Surface-Enhanced Raman Spectroscopy (SERS) Detection

SERS is an ultrasensitive, nondestructive, and noninvasive spectroscopic technique for the detection of molecules on or near the surface of plasmonic nanostructures [146]. As another ultrasensitive label-free analytical tool, SERS has also been successfully applied in the detection of mycotoxins. For instance, Galarreta et al. [147] prepared a metallic nanostructure 2D SERS platform on a glass coverslip surface by electron beam lithography, and embedded it into a PDMS microfluidic channel for the detection of OTA. An HS–aptamer of OTA was immobilized onto the metallic nanostructure. When OTA was present in the sample, the Raman spectrum of the OTA–aptamer complex was subsequently acquired. Additionally, Li et al. [148] constructed a SERS aptasensor chip based on exonuclease-assisted recycling amplification for the ultrasensitive determination of AFB1 in spiked peanuts. When AFB1 is present in the sample, AFB1 aptamers will be released from the hybridization duplex strands of AFB1 aptamers and their partial complementary strands, and immediately hybridized with hairpin DNA on the Au film of chip surface. Then the hairpin DNA was hydrolyzed by exonuclease III at a restriction site, leaving short single-stranded DNA to capture Raman tags via hybridization on Au film and releasing complementary DNA for recycling. Hence, a large number of Raman probes were anchored on the Au film of the chip. High sensitivity and good selectivity for the detection of AFB1 were obtained. The linear range and LOD were determined to be 1 × 10^−6^–1 μg/L and 0.4 × 10^−6^ μg/L, respectively. These aptamer–SERS sensing chips provide an important application for more rapid and portable determination of mycotoxins.

#### 3.4.4. Optical Waveguide Lightmode Spectroscopy (OWLS) Detection

OWLS, another highly sensitive, rapid label-free detection method, has also been applied successfully to detect the interaction between antigen and antibody. OWLS uses evanescent field for the in situ study of surface processes at a molecular level. So far, there have been few reports about the development of an OWLS sensor chip for the detection of mycotoxins. Majer-Baranyi et al. [149] applied direct and indirect immunoassays using DON polyclonal antibodies in OWLS sensor chips for the detection of DON. The direct OWLS immunochip showed an unstable sensor response and a low sensitivity above 1 μg/L for DON in a real sample, not being sufficient for DON determination. In contrast, the competitive OWLS immunochip provided reproducible quantitative detection in the concentration range of 5–5 × 10^4^ μg/kg for DON spiked in wheat flour. Recently, they also detected AFB1 in spice paprika samples using the same method, with an LOD of 0.35 μg/kg [150]. In addition, Adányi et al. [151] also developed an OWLS immunochip for determining AFB1 and OTA in barley and wheat flour samples using competitive and direct immunoassays. All of the results showed that OWLS immunochips have the potential for rapid, highly sensitive detection of mycotoxins in foods.

#### 3.4.5. Broad-Band Mach–Zehnder Interferometry (BB-MZI) Detection

SPR, SERS, and OWLS sensors have excellent sensitivity, but they need external optical components, especially laser sources for excitation. Hence, these label-free detection methods need expensive and large-scale instruments that make them appropriate to be used only in the lab. In order to move detection methods from the lab to the field, Pagkali et al. [152] developed a novel, regenerated, reused label-free immunochip based on monolithically integrated arrays of Broad-Band Mach–Zehnder Interferometry (BB-MZI) along with their respective light-emitting diodes (LEDs) for the detection of OTA in beer. An OTA–OVA conjugate was immobilized on the sensing arm area of BB-MZI. The LOD of OTA in beer was 2.0 μg/L, and the dynamic detecting range was 4–100 μg/L. The miniaturized biosensor chip is suitable for the development of a portable instrument for point-of-need determination.

#### 3.4.6. Giant Magnetoresistive Detection

Here, Mak et al. [153] integrated the sandwich immunoassay into a new multiplex magnetic nanotag-based biochip detection platform for the simultaneous detection of multiple mycotoxins including AFB1, ZEA, and HT-2. The sensitivity can be up to sub-picomolar concentration level, and the LOD was 0.05 μg/L. Compared to the fluorescent labels, magnetic nanotags (MNTs) can be detected with low-cost giant magnetoresistive (GMR) sensors such as spin-valve. The GMR biosensor chip system has the capacity for low-cost, real-time, and rapid detection of multiplex mycotoxins.

## 4. Conclusions

In conclusion, microchips, including microfluidic chips and microarray chips, can be employed in mycotoxin determination in foods by optical detection, electrochemical detection, photo-electrochemical detection, or a label-free detection method. A microchip is an innovative method that could convert conventional methods into more efficient micro-scale devices due to its unique properties of automation, integration, portability, high throughput, low sample consumption, and rapid sensing time. However, most microchips applied to mycotoxin determination are used only in the lab due to the large-scale detecting instruments. Hence, one of the major challenges of microchips for the detection of mycotoxins is to minimize the detecting instruments in order to move detection methods from the lab to the field. Additionally, the complexity of sample matrices, the diversity of mycotoxins, and the electrostatic force of microchip channels are also major challenges in the use of microchips for the detection of mycotoxins. Hence, sample pretreatment is essential before biochip analysis for high-sensitivity and -specificity detection. So far, most of the sample pretreatments were realized off-chip. So, scientists have to exert more effort and spend more time on the realization of on-chip sample pretreatments. Furthermore, in view of the diversity of mycotoxins, multiplex detection remains a goal for the future. Meanwhile, in order to avoid the sample adsorption that was caused by the electrostatic force of the microchips, new chip materials without adsorption property, or physical adsorption coating and chemical bonding coating, need to be developed. Efforts should focus on the integration of other separation methods and detection techniques into the biochip systems. Paper-based biochips, regenerable biochips, and multi-channel biochips will be popular in the determination of mycotoxins. The commercial application of microchip devices is the ultimate goal for the detection of mycotoxins in foods.

## Figures and Tables

**Table 1:** The representative mycotoxins and their toxicities.

Mycotoxins		Abbreviation	Toxicities
AFs	Aflatoxin B1	AFB1	AFs play an important role in developing countries; they have acute toxicity and can lead to cancer, immunologic suppression, and nutritional interference [25,26,27]. AFB1 is the most potent carcinogenic agent [28]. AFB1 and AFM1 are classified as group 1 carcinogens by the International Agency for Research on Cancer (IARC) [29,30].
	Aflatoxin B2	AFB2	
	Aflatoxin M1	AFM1	
	Aflatoxin G1	AFG1	
	Aflatoxin G2	AFG2	
Alternaria toxins	Alternariol	AOH	AOH and AME have no acute toxic effects, but possess carcinogenicity, with an especially high incidence of esophageal cancer [31]. Moreover, they also display mutagenicity, genotoxicity, and cytotoxicity, and can induce DNA breaks and inhibit the activity of topoisomerase [32,33,34].
	Altenariol monomethyl ether	AME	
	Tenuazonic acid	TeA	TeA has acute toxicity and was listed in the Food and Drug Administration (FAD) toxic chemical register [32,35]; it inhibits protein synthesis [36] and has cytotoxic [37], carcinogenic, and synergistic effects [38].
Ochratoxin A		OTA	OTA has nephrotoxic, hepatotoxic, neurotoxic, teratogenic, and immunotoxic effects [39,40], and is classified as a Group 2B carcinogen [41].
Deoxynivalenol		DON	DON displays cytotoxicity [42,43] and can induce emesis, anorexia and diarrhea, weight loss, neuroendocrine changes, immunological effects, leukocytosis, hemorrhaging, or circulatory shock [44].
Zearalenone		ZEA	ZEA displays carcinogenicity, immunotoxicity, genotoxicity, reproductive and developmental toxicity; in addition, it has an effect on the endocrine system [16,45,46].
Patulin		PTL	PTL displays neurotoxicity, embryotoxicity, teratogenicity, immunotoxicity, and can cause convulsions, dyspnea, pulmonary congestion, edema, ulceration, hyperemia, and distension of the gastrointestinal tract [47,48].
T-2 toxin		T-2	T-2 is a potent inhibitor of protein synthesis and mitochondrial function, and shows immunosuppressive and cytotoxic effects [49]; it also has extremely toxic effects on the skin and mucous surfaces [50,51].
Fumonisin B1		FB1	FB1 can lead to hepatotoxicity, cancer, and apoptosis [52,53], and can cause pulmonary edema and hydrothorax in pigs [54]. FB1 is classified as a Group 2B carcinogen [41].
Citrinin		CIT	CIT displays reproductive toxicity, as well as nephrotoxic, embryotoxic, teratogenic, hepatotoxic, immunotoxic, and carcinogenic properties [55,56,57].

**Table 2:** Representative materials used for the fabrication of microchips.

Materials		Process	Advantages	Disadvantages
Inorganic materials	Silicon [68]	Standard photolithography	Resistance to organic solvents, high thermoconductivity, simple metal deposition, stable electroosmotic mobility.	Fragile, opaque, poor electrical insulation, hardness, high cost, time-consuming, labor-intensive, the bonding is difficult and requires a sterile environment.
	Glass [69]	Standard photolithography	Optically transparent and electrically insulating, resistance to organic solvents, high thermoconductivity, simple metal deposition, stable electroosmotic mobility, easy surface modification.	Fragile, high cost, time-consuming, labor-intensive, bonding is difficult and requires a sterile environment.
Elastomeric polymers	PDMS [70]	Cast molding	Optically transparent, high elasticity, low cost, easy and reversible binding, non-toxicity, permeability, compatible for cell culture, could integrate with the micropump and microvalve.	Could not withstand high temperatures, low thermoconductivity, strong nonspecific adsorption, poor organic solvent compatibility.
Rigid plastic polymers	PMMA [71]	Thermal molding	Organic solvent compatibility better than PDMS, low cost, can produce thousands of replicas at a high rate, the thermal bonding does not require a sterile environment.	Poor permeability and heat conductivity, high rigidity, difficult surface modification, cannot withstand high temperatures.
	PI [72]			
	PC [73]			
	PS [74]			
	PET [75]			
	PVC [76]			
	COC [77]			
	Teflon PFA [78]		Extremely inert to chemical solvents, optically transparent, moderate permeability, antifouling, proper mechanical strength, low nonspecific absorption, no leaching of residue molecules from the material bulk into the solution in the channel.	Melting temperatures are high (over 280 °C).
	Teflon FEP [78]			
Hydrogel polymers	PEG [79,80]	UV-induced polymerization	Highly porous with controllable pore sizes, allowing small molecules or even bio-nanoparticles to diffuse through, compatible for cell culture, short preparation time.	The bonding is difficult.
Photosensitive polymer	SU-8 photoresist [81]	Photolithography	Stable even at high temperatures, resistant to most solvents, and optically transparent.	High cost, high stiffness, poor permeability, and non-uniform thickness.
	NOA81 [82]		Transparent, rapid, solvent-resistant, lower auto-fluorescence, and the thickness can be easily manipulated.	
	pCLLA [83]		Non-toxicity, biocompatibility, biodegradability, rapidness, and flexibility in materials processing.	
Paper	Paper [84]	Lithographic methods and printing (cutting) methods	Portable and low-cost analysis, without the need for power or external components; large surface-to-volume ratio, the cheapest materials.	Liquids may not be well confined in the channel due to hydrophobicity, the applicable detection methods are relatively limited, low detection sensitivity, evaporation of liquid.

**Table 3:** Microchips integrated various kinds of detection methods for detecting mycotoxins.

Target Analyte	Detection Method	Characteristic of the Microchip	Real Sample	LOD	Reference
OTA	Fluorescence detection	PDMS microfluidic chip based on aqueous two-phase extraction	Red wine	0.26 μg/L	[22]
AFB1	Fluorescence detection	Smectite-PAM nanocomposite based on strip microfluidic sensor chip	Corn	6.09 μg/kg	[86]
FB1	Fluorescence detection	Microarray immunochip based on synthetic mimotopes	Maize and wheat	11.1 μg/L	[89]
OTA	Fluorescence detection	Microarray immunochip	Cereals	3.8–100 μg/kg	[90]
			Coffee	7 μg/kg	
			Wine	38 μg/kg	
DON	Fluorescence detection	Microarray immunochip	Oats	0.2–50 μg/kg	[91]
			Effluent	4 μg/L	
AFB1	Fluorescence detection	Protein microarray immunochip	Drinking water	0.01 μg/L	[92]
AFM1				0.24 μg/L	
DON				15.45 μg/L	
OTA				15.39 μg/L	
T-2				0.05 μg/L	
ZEA				0.01 μg/L	
AFB1	Fluorescence detection	High throughput biochip based on photonic crystal microsphere (PHCM) suspension array	Cereal samples	15.96 × 10^−6^ μg/L	[93]
OTA				3.96 × 10^−6^ μg/L	
FB1				0.011 μg/L	
OTA	Chemiluminescence detection	Indirect competitive immunochip	Green coffee	0.3 μg/L	[96]
AFs, OTA, DON, FB1	Chemiluminescence detection	Regenerable microarray immunochip	Oat extracts	/	[97]
OTA	Colorimetric detection	Combination of an OTA-responsive hydrogel with a distance-based readout V-chip	Beer	0.51 μg/L	[98]
AFB1	Colorimetric detection	Combination of an AFB1-responsive hydrogel with a distance-based readout V-chip	Beer	0.55 μg/L	[99]
AFB1	Colorimetric detection	Integrated, smartphone-app-chip (SPAC) system	Corn	3 μg/kg	[100]
AFB1	Electrochemical detection	96-well screen-printed microarray immunochip	Corn	0.03 μg/L	[103]
AFM1	Electrochemical detection	Gold microelectrode array immunochip	Milk	0.008 μg /L	[104]
CIT	Electrochemical detection	Microfluidic electrochemical immunochip	Rice	0.1 μg/L	[105]
DON	Electrochemical detection	Microfluidic chip coupled with gold microelectrode arrays	Wheat	6.25 μg/L	[106]
AF	Electrochemical detection	Microfluidic chip coupled with gold microelectrode arrays	Foods	0.08–0.65 μg/kg	[107]
ZEA	Electrochemical detection	Microfluidic chips coupled with magnetic bead-based immunoassay	Baby foods	1 μg/kg	[108]
ZEA	Electrochemical detection	Microfluidic chips coupled with magnetic bead-based immunoassay	Infant foods	0.4 μg/L	[109]
ZEA	Electrochemical detection	Microfluidic chips coupled with magnetic bead-based ELISA	Feedstuffs	0.41 μg/L	[110]
OTA	Electrochemical detection	Microfluidic chips coupled with magnetic bead-based ELISA	Apples	50 μg/kg	[111]
OTA	Photo-electrochemical detection	Chemiluminescence-based ELISA in microfluidic chip with integrated photodiodes	Beer	0.1 μg/L	[115]
			Red wine	2 μg/L	
OTA	Photo-electrochemical detection	Regenerable chemiluminescence-based immunoassay in microfluidic chip with integrated photodiodes	Red wine	<2 μg/L	[116]
OTA, AFB1 and DON	Photo-electrochemical detection	Microfluidic multiplexed biosensor chip with a permanent magnet valves	/	/	[117]
OTA	Photo-electrochemical detection	Aptamer-based sandwich assay in a multichannel microfluidic chip	Beer	0.82 μg/L	[118]
AFB1	MS	Plastic microfluidic chip coupled with ESI-MS	/	/	[119]
AFs	MS	Microfluidic chip-based nano LC coupled with QqQ-MS	Peanut	0.004–0.008 μg/kg	[121]
AFB2	SPR	Microchip SPR immunochip using anti-AFB2 scFv antibody	Almond	0.9 μg/L	[124]
ZEA	SPR	Microchip SPR immunochip using anti-ZEA scFv antibody	Sorghum	7.8 μg/L	[125]
NIV	SPR	Microchip SPR immunochip using monoclonal antibody	Wheat	200 μg/kg	[126]
DON				100 μg/kg	
T-2 and HT-2	SPR	Microchip SPR immunochip using monoclonal antibody	Baby food and breakfast cereal	25 μg/kg	[127]
			Wheat	26 μg/kg	
T-2 HT-2	SPR	Microchip SPR immunochip using monoclonal antibody	Wheat	31 μg/kg	[128]
			Breakfast cereal	47 μg/kg	
			Maize-based baby food	36 μg/kg	
DON			Wheat	12 μg/kg	
			Breakfast cereal	1 μg/kg	
			Maize-based baby food	29 μg/kg	
PTL	SPR	Microchip SPR immunochip using polyclonal mono-specific antibodies	/	15.41 μg/L	[129]
DON	iSPR	Microchip multiplex microassay iSPR immunochip	Maize	84 μg/kg	[130]
			Wheat	68 μg/kg	
ZEA			Maize	64 μg/kg	
			Wheat	40 μg/kg	
DON	iSPR	Microchip nanostructured iSPR immunochip	Beer	17 μg/L	[131]
OTA				7 μg/L	
DON	iSPR	Microchip benchtop SPR (Biacore) with two separates nanostructured iSPR immunochip	Barley	26 μg/kg	[132]
ZEA				6 μg/kg	
T-2				0.6 μg/kg	
OTA				3 μg/kg	
FB1				2 μg/kg	
AFB1				0.6 μg/kg	
DON	SPR-MS	Coupled SPR immunochip and ambient ionization MS	Beer	/	[133]
AFB1	iSPR	AuNP-enhanced iSPR immunochip	Spiked peanut	0.008 μg/L	[134]
OTA				0.03 μg/L	
AEN				0.015 μg/L	
OTA	SPR	AuNP-enhanced SPR immunochip	Red wine	0.068 μg/L	[135]
AFM1	SPR	AuNP-enhanced SPR immunochip	Milk	0.018 μg/L	[136]
AFB1	SPR	SPR immunochip based on GBP-ProG crosslinker	Buffer and corn extracts	1000 μg/L	[137]
OTA	SPR	SPR microchip based on aptamer	Wine and peanut oil	0.005 μg/L	[138]
AFB1	SPR	SPR microchip based on aptamer	Red wine	124.91 μg/L	[139]
OTA	SPR	Localized SPR microchip based on aptamer	Ground corn samples	403 μg/L	[140]
OTA	SRP	SPR-polarization microchip based on aptamer	/	0.005 μg/L	[141]
DON	SPR	SPR microchip based on MIP	DON standard solution	>1 μg/L	[142]
ZEA	SPR	SPR microchip based on MIP	Corn	0.3 μg/kg	[143]
CIT	SPR	SPR biosensor chip based on MIP	Red yeast rice	0.0017 μg/L	[144]
T-2	SPR	SPR microchip based on nanopatterned π-conjugated MIP	/	0.47 × 10^-4^ μg/L	[145]
OTA	SERS	Microfluidic chip embedded 2D SERS platform	/	/	[147]
AFB1	SERS	SERS aptasensor chip based on exonuclease-assisted recycling amplification	Spiked peanuts	0.4 × 10^−6^ μg/L	[148]
DON	OWLS	Immunochip based on OWLS	Wheat flour	/	[149]
AFB1	OWLS	Immunochip based on OWLS	Spice paprika samples	0.35 μg/kg	[150]
AFB1, OTA	OWLS	Immunochip based on OWLS	Barley and wheat flour samples	/	[151]
OTA	BB-MZI	Si immunochip based on monolithically integrated BB-MZI	Beer	2.0 μg/L	[152]
AFB1, ZEA, HT-2	GMR	Multiplex magnetic nanotag-based biochip	/	0.05 μg/L	[153]
